# Gesetzliche Neuregelung der „Kinderpornographie“ (§ 184b StGB)

**DOI:** 10.1007/s00115-023-01455-x

**Published:** 2023-03-03

**Authors:** Harald Dreßing

**Affiliations:** Klinik für Psychiatrie und Psychotherapie, Zentralinstitut für Seelische Gesundheit, Medizinische Fakultät Mannheim, Universität Heidelberg, J 5, 68159 Mannheim, Deutschland

## Hintergrund

Im Juli 2021 trat eine neue Fassung des § 184b StGB in Kraft, die eine erhebliche Verschärfung des Strafrahmens für die Verbreitung, den Erwerb und den Besitz kinderpornographischer Inhalte vorsieht. Bisher häufiger praktizierte Lösungen in außergerichtlichen Verfahren oder die Anordnung von Bewährungs- oder Geldstrafen dürften nach den verschärften Bedingungen des § 184b StGB seltener zur Anwendung kommen, da bereits der Besitz einer einzigen kinderpornographischen Datei grundsätzlich ein Verbrechen darstellt, das mit einer Mindestfreiheitsstrafe von einem Jahr strafbewehrt ist und die Staatsanwaltschaft in solchen Fällen zwingend Anklage erheben muss. Diese gesetzliche Neuregelung bedingt nicht nur Änderungen bei den Strafverfolgungsbehörden und den Gerichten, sondern erfordert auch eine intensivere Befassung von Psychiatrie, Psychotherapie und forensischer Psychiatrie mit der Thematik „Kinderpornographie“. Relevant ist dabei z. B. die Frage, ob der Besitz kinderpornographischer Inhalte unter Umständen auch eine Vorstufe für pädosexuelle Hands-on-Delikte sein kann. Sexuelle Gewalt gegen Kinder kann in vielen Formen begangen werden und pädosexuelle Delikte kommen auch in unterschiedlichen Kombinationen vor. Prognostische Fragesellungen werden insoweit an unser Fachgebiet herangetragen werden, wenn sich die Gerichte zukünftig häufiger mit Kinderpornographie befassen werden.

## Terminologie

Der in der juristischen Diktion verwendete Begriff der „Kinderpornographie“ im Zusammenhang mit Kinderbildern, die sexualisierte Gewalthandlungen zum Inhalt haben, ist zumindest aus psychiatrischer Sicht nicht unproblematisch, stellt er doch einen Bezug zu der nichtkriminellen Pornographie im Erwachsenenbereich her, in dem entsprechendes Bildmaterial in der Regel einvernehmlich produziert wird. Die Anfertigung „kinderpornographischer“ Bilder beinhaltet aber eo ipso eigentlich die Ausübung sexualisierter Gewalt, denn Kinder unter 14 Jahren können unter Zugrundelegung der in Deutschland geltenden gesetzlichen Normen in die Produktion solcher Bildaufnahmen nicht wirksam einwilligen. Insoweit impliziert dann auch der Besitz und der Konsum solchen Bildmaterials die Einwilligung in die mit sexualisierter Gewalt verbundenen Entstehungsbedingungen solcher Fotos oder Videos. In der wissenschaftlichen Literatur wird der Begriff „Kinderpornographie“ deshalb auch kritisch diskutiert, und es wird für diese Thematik in einigen Publikationen eine andere Terminologie vorgeschlagen. So werden in Publikationen zu diesem Thema Begriffe wie z. B. „sexual abuse images in cyberspace“ verwendet und das inkriminierte Bildmaterial wird als „indecent images of children (IIOC)“ bezeichnet [9]. Auch wenn es insoweit also angemessener wäre z. B. von Missbrauchsabbildungen von Kindern zu sprechen, wird man zumindest im forensisch-psychiatrischen Alltag um den Begriff der Kinderpornographie nicht herumkommen. Dabei sollte einem die Bedeutung sprachlicher Konventionen aber immer bewusst sein und die terminologische Problematik sollte von PsychiaterInnen in allen Kontexten auch thematisiert werden, um kognitiven Verzerrungen von pädosexuellen StraftäterInnen keinen Vorschub zu leisten. Der Begriff der Kinderpornographie könnte bei Beschuldigten die Vorstellung einer vermeintlichen Einwilligung der Kinder bei der Herstellung des Bildmaterials fördern. Aus der gutachtlichen Praxis sind Fälle bekannt, in denen der Besitz, der Konsum und auch die Weiterverbreitung von Bildmaterial, das sexualisierte Gewalt gegen Kinder zum Inhalt hat, von den Beschuldigten gar nicht als Straftat wahrgenommen wird. Die Tatsache, dass man mit Besitz und Konsum solcher Bilder zu deren kriminellen Entstehungsbedingungen beiträgt, wird in den Einlassungen von den Beschuldigten nicht selten abgestritten und verdrängt, oder es werden groteske Begründungen vorgebracht, dass entsprechendes Bildmaterial – oft in erheblichem Umfang – durch versehentliche Klicks auf den Rechner gelangt sei.

## Risikoprofil bei den Beschuldigten

Die Auswirkungen des Besitzes und Konsums von Missbrauchsabbildungen von Kindern insbesondere im Hinblick auf mögliche Hands-on-Delikte werden in der wissenschaftlichen Literatur kontrovers diskutiert. Bisherige Studien konnten eine direkte Auswirkung auf andere pädosexuelle Straftaten nicht belegen [5]. Entsprechende Taten werden oft als voyeuristischer Natur rationalisiert und als scheinbar weniger gefährlich eingeschätzt als z. B. die Herstellung oder Verbreitung entsprechender Bilder oder andere pädosexuelle Verhaltensweisen [2].

Eine mögliche katalytische Wirkung des Konsums entsprechenden Bildmaterials ist als Hypothese aber auch nicht von der Hand zu weisen. In der öffentlichen Wahrnehmung werden Delikte im Zusammenhang mit dem Besitz und Konsum von Missbrauchsabbildungen als gravierende Straftaten wahrgenommen. In einer aktuellen Untersuchung zeigte sich, dass in der Allgemeinbevölkerung solche Delikte als schwerwiegender als die meisten anderen Straftaten eingeschätzt werden, wobei die Risiken im Zusammenhang mit Rückfällen und Hands-on-Delikten eher überschätzt werden [14].

In einer Metaanalyse von 21 Studien mit insgesamt 4464 Online-Sexualstraftätern zeigte sich, dass bei jedem achten auch ein bekanntes Hands-on-Sexualdelikt zum Zeitpunkt der jüngsten Verurteilung bekannt war. Darüber hinaus zeigte sich in den Studien, die auch selbst berichtete Daten zur sexuellen Vorgeschichte erhoben hatten, dass 55 % der Online-Sexualstraftäter auch über ein Hands-on-Delikt berichteten [11]. Die Ergebnisse dieser Metaanalyse verdeutlichen, dass die Auswahl der Stichproben, die Erhebungsmethoden und die Beobachtungszeiträume einen wesentlichen Einfluss auf die Ergebnisse haben. Diese Prämissen sind bei der Interpretation der im Folgenden skizzierten Studien zu beachten. So fand sich in einer Stichprobe von 231 Männern, die wegen des Konsums von illegalem Bildmaterial angeklagt wurden, in einem Beobachtungszeitraum von 6 Jahren, dass der alleinige Konsum kein Risikofaktor für die Begehung von Sexualdelikten ist, zumindest nicht für jene Personen, die davor noch nie ein Sexualdelikt begangen hatten. Die Mehrheit der untersuchten Männer in dieser Stichprobe hatte keine Vorstrafen wegen Hands-on-Delikten und die Prognose war sowohl für Hands-on- wie Hands-off-Delikte günstig [6]. Ähnliche Ergebnisse ergab eine Studie, die eine Stichprobe von Tätern mit ausschließlichem „Kinderpornographiekonsum“ mit einer Stichprobe von Tätern verglich, die sowohl durch „Kinderpornographiekonsum“ als auch durch Hands-on-Delikte auffällig wurde. Die gesamte Rückfallquote in dieser Studie betrug 12,6 % für Wiederverurteilungen wegen einer Sexualstraftat nach einer durchschnittlichen Nachbeobachtung von 13 Jahren, aber nur 2,7 % der Täter mit ausschließlichem „Kinderpornographiekonsum“ wurden wegen eines nachfolgenden Kontaktdelikts verurteilt [8]. Ergebnisse einer weiteren Metaanalyse legen nahe, dass sich Täter mit ausschließlichem „Kinderpornographiekonsum“ von Tätern unterscheiden, die Hands-on-Delikte begehen, wobei Täter, die wegen „Kinderpornographiekonsums“ auffällig werden und zusätzlich Hands-on-Delikte begehen häufiger als pädophil zu klassifizieren sind und ein besonderes Risiko darstellen [1].

Bei einem Beobachtungszeitraum von 5 Jahren fand sich in einer Stichprobe von 266 männlichen „Kinderpornographie“-Straftätern bei 3 % eine neue Kontaktsexualstraftat gegen ein Kind und bei 9 % eine neue „Kinderpornographie“-Straftat. Folgende Risikofaktoren konnten dabei ermittelt werden: jüngeres Straftäteralter, andere Vorstrafen, sexuelle Kontaktdelikte in der Vorgeschichte, Bewährungsversagen, sexuelles Interesse an kinderpornographischem Material insbesondere an der Darstellung von Jungen [13].

In einer aktuellen Studie konnte gezeigt werden, dass bei der Einschätzung der Auswirkungen des Konsums von „Kinderpornographie“ komplexe Wechselwirkungen zu bedenken sind [10]. Diese Studie untersuchte die Zusammenhänge verschiedener Arten von Pornographiekonsum einschließlich des Konsums von „Kinderpornographie“, kriminogenen Kognitionen und paraphilen Interessen bei sexuellen Straftaten gegen Jungen oder Mädchen. Dabei zeigten multivariate Analysen, dass der Konsum von „Kinderpornographie“ das Risiko sexueller Straftaten zum Nachteil von männlichen Kindern und Jugendlichen erhöht. Auch in einer Post-hoc-Analyse männlicher Sexualstraftäter zeigte sich eine signifikante Assoziation zwischen „Kinderpornographiekonsum“ und selbst berichteten pädosexuellen Hands-on-Delikten [3].

Weitere empirische Forschung ist auf diesem Gebiet unbedingt nötig, da die Zahl der Verurteilungen wegen „Kinderpornographie“ in den letzten Jahren stetig ansteigt [7].

Die bisherigen empirischen Studien weisen weitgehend übereinstimmend darauf hin, dass sich „Online-Sexualstraftäter“ von „Kontakt-Sexualstraftätern“ und von Tätern, die beide Arten von Sexualstraftaten begehen, unterscheiden. Rückfälle bei „reinen Online-Sexualstraftätern“ sind aufgrund des Mangels an umfangreicheren empirischen Studien und des anzunehmenden großen Dunkelfeldes schwer zu beurteilen [4].

Grundsätzlich sind bei Therapieplanung und gutachtlicher Einschätzung die möglichen Motive der Beschuldigten zu beachten. Nicht jeder Konsum von „Kinderpornographie“ ist durch pädophile sexuelle Interessen motiviert, sondern kann z. B. auch Manifestation von hypersexuellen oder zwanghaften Verhaltensweisen sein. Für die Motivanalyse sind deshalb Kenntnisse u. a. über Muster und Häufigkeit des Konsums ebenso wichtig wie die Analyse des Bildmaterials im Hinblick auf Geschlecht, Alter und die Eindeutigkeit sexualisierter Darstellungen von Kindern [12].

## Fazit für die Praxis

Therapieplanung und prognostische Einschätzung bei Konsumenten von „Kinderpornographie“ erfordern eine differenzierte Einzelfallbeurteilung. Bisherige Studien weisen auf ein moderates Rückfallrisiko hin, wobei ein hohes Dunkelfeld anzunehmen ist und die Wahrscheinlichkeit für Hands-on-Delikte beim Vorliegen weiterer Risikofaktoren erhöht ist.
